# Development of a program theory for shared decision-making: a realist synthesis

**DOI:** 10.1186/s12913-019-4649-1

**Published:** 2020-01-23

**Authors:** Tamara Waldron, Tracey Carr, Linda McMullen, Gill Westhorp, Vicky Duncan, Shelley-May Neufeld, Lori-Ann Bandura, Gary Groot

**Affiliations:** 1 Department of Community Health and Epidemiology, Health Sciences Building, 107 Wiggins Road, Saskatoon, SK S7N 5E5 Canada; 2 Department of Psychology, University of Saskatchewan Arts, 154, 9 Campus Drive, Saskatoon, SK S7N 5A5 Canada; 3 Charles Darwin University, Ellengowan Drive, Casuarina, NT 0810 Australia

**Keywords:** (3–10 words): shared decision making, Realist review, Mechanisms, Quality improvement, Health systems, Medical decision making

## Abstract

**Background:**

Shared Decision-making (SDM), a medical decision-making model, was popularized in the late 1980s in reaction to then predominate paternalistic decision-making, aiming to better meet the needs of patients. Extensive research has been conducted internationally examining the benefits of SDM implementation; however, existing theory on *how* SDM works, for *whom,* in which *circumstances*, and *why* is limited. While literature has shown positive patient, health care provider, and system benefits (SDM outputs), further research is required to understand the nuances of this type of decision-making. As such, we set out to address: “In which situations, how, why, and for whom does SDM between patients and health care providers contribute to improved engagement in the Shared Decision-making process?”

**Methods:**

To achieve our study goals we conducted a seven-step realist synthesis process, which included: (1) preliminary program theory development, (2) search strategy development, (3) selection and appraisal of literature in accordance with realist methodology, (4) data extraction, (5) identification of relevant formal theories, (6) data analysis and synthesis, and (7) formation of a revised program theory with the input of stakeholders. This process was done in accordance with RAMESES guidelines and publication standards for a realist synthesis. Expert consultations were also held to ensure consistency within the SDM literature.

**Results:**

Through our realist synthesis, we developed a program theory of SDM which includes three contexts (pre-existing relationship, difficulty of decision, and system support), eight mechanism sets (anxiety, trust, perception of other party capacity, perception of time, self-efficacy, world view, perception of capacity to external support, and recognition of decision), and one outcome (engagement in SDM).

**Conclusions:**

As far as the authors of this paper are aware, this paper is the first to begin unpacking *how* SDM works, for *whom*, in which *circumstances*, and *why.* By examining key mechanism sets and exploring how they facilitate or inhibit SDM, we have produced a program theory that may assist health care professionals, policy makers, and patients. While further research is suggested to further unpack the concepts identified within this paper, this provides an initial understanding into the theory behind SDM.

**Registration:**

PROSPERO: CRD42017062609.

## Background

### Shared Decision-making

Shared Decision-making (SDM) is a style of decision-making involving health care providers (HCPs) and patients, with the aim of making a joint informed and patient-centred decision [1, 2]. Since SDM was first introduced in literature in the late 1980s to early 1990s [1, 3], certain elements have been identified as essential, including: involvement of, at minimum, the physician and patient [4–7], a balanced relationship between HCPs and patient, exchange of information including patients’ values and preferences [8–10], discussion of options [1, 8, 11], and a mutually agreed upon decision [1]. When combined, these elements are thought to create an environment for patients and HCPs that fosters and encourages open communication, evidence-based decision-making, greater patient involvement in the health care process, and ultimately optimal patient-centered care.

SDM can be especially useful in complex cases where multiple options exist [12], such as the decision-making required when dealing with a cancer diagnosis. Cancer diagnoses require multiple high stakes decisions to be made in a narrow window of time, and often with incomplete evidence. Consequently, oncology patients frequently need increased support during their decision-making process [13–15]. Emerging technologies and treatments increase potential options creating more decisional conflict and anxiety for patients [16], and an even greater need for support.

Two key elements of SDM are the elicitation of patient preferences and knowledge exchange between parties [8–10, 17, 18]. When patients are consulted, the vast majority (92%) [19] desire physicians to explain all potential options, elicit their preferences, and engage them in preference and knowledge exchange to some extent [19, 20]. Furthermore, patients expect their HCPs to explain treatment benefits and risks specific to their individual scenario [21]. Those who are more likely to desire SDM include: younger patients [5, 19, 22], females [5, 20, 22], and/or those with a severe diagnosis [5, 23]. In contrast to patient desires/preferences, previous studies have found only 39% of patients felt that SDM occurred in their consultations [21], and 37% were involved less than they wished to be [24]. SDM is able to increase involvement congruency between patient expectations and reality by eliciting patient preferences and values [18, 25, 26]. While empirical research has been valuable to inform the development of several frameworks of SDM, the absence of theory to explain how SDM works, for whom, and in what contexts has limited efforts for creating an implementable version of SDM outside the research environment. While patient involvement itself can assist to increase informed decision-making, it does not ensure a patient-centered decision. A patient may be included within the discussion but lack meaningful engagement (such as the elicitation of personal values that may influence treatment preferences). It is important for patients to be provided with tailored information and in a manner they are able to comprehend. Use of SDM allows for patients to make both an informed and patient-centered decision [1]. The project described in this paper is required to understand how the process of SDM allows for a patient-centered, informed decision. The results of this research will allow the generation of testable hypotheses of SDM for which future research may create a predictive understanding of how, when, and for whom SDM works.

SDM literature focuses on descriptive frameworks/models and conceptual development [5, 27, 28], and empirical implementation [29, 30]. Current literature struggles to synthesize existing SDM research [31]. Previous authors have noted that current evidence focusing on empirical measures of SDM lack an association to patient and health outcomes [31]. This has resulted in a gap between existing frameworks and empirical SDM research, where empirical research does not reflecting current SDM theories [32]. Prior research has indicated certain factors inhibit and/or promote SDM [9, 33, 34]; however, why and how these factors work is unclear. For example, Shepherd and colleagues identified factors that may inhibit physicians from implementing SDM, such as time constraints and information exchange, but they do not explain *how or why* this inhibits the process [34] (e.g. does the perception of a time constraint inhibit physicians from implementing SDM due to fee-for-service structures, patient case-loads, or another reason?). While factors that facilitate and hinder SDM have been identified, research fails to connect these factors to relevant contexts. Without understanding contexts, we are unable to assess the impact of such factors in a research or clinical environment. As current literature does not explore how or why inhibiting and promoting factors impact SDM, individuals attempting to implement SDM [29, 35] often struggle to use this process successfully potentially due to the ambiguity regarding how such factors influence implementation.

Several models and frameworks exist within literature. Two well cited models include: the “three-talk” model [36] (original article cited 941 times) and the Ottawa Decision Support Framework (ODSF) [37] (cited 477 times). The original “three-talk” model discusses three stages of a decision (choice talk, option talk, and decision talk) to elicit key components of SDM such as value and knowledge exchange and addressing decisional uncertainty [36, 38, 39]. At the time our study began, this model had yet to emphasize additional people beyond the patient-physician dyad. However, it has recently been updated to change “choice talk” to “team talk”, better incorporating other individuals being involved [39, 40]. The second framework, ODSF, aims to improve decisional quality through the use of tools aimed to better prepare patients and HCPs for SDM. ODSF presents key facets of SDM that must be met for a quality decision to be made [5, 41]. However, this framework has yet to formalize the involvement of supports.

A third model, the Interprofessional-SDM model (IP-SDM), is well-cited (1063 citations) and respected in the field [17, 33, 42–46]. This model has gained significant attention, including a full issue within the Journal of Interprofessional Care [43]. This model recognizes that the medical decision process is broader than the traditional patient-physician dyad, expanding to include other HCPs and patient supports, such as family and friends. IP-SDM presents several “steps” in a fluid and iterative process, including: identifying a decision to be made, information exchange, values/preferences exchange, assessing feasibility, discussion of preferred choice, selecting the actual choice, and treatment implementation [17]. The fluidity acknowledged in this model allows for the natural “back-and-forth” that occurs during a decision-making process. IP-SDM presumes that the dynamic exchange that occurs between patients and HCPs, including the elicitation of values and preferences, will result in a patient-centered decision. The breadth of this model is translated by the numerous fields it has been applied to, stretching beyond medical consultations [43]. However, the current postulation of the framework requires expansion to form testable hypotheses. While literature has specifically identified the need to connect a theory to the IP-SDM model [43], all three of the above models lack a testable theory.

In the absence of an explicit theory to explain how SDM works, for whom and in which circumstances, we used the IP-SDM model as the basis upon which to build such a theory.

### Review purpose

The purpose of this research was to develop a realist program theory for SDM. To meet this purpose, we conducted a realist synthesis, selected because it seeks to understand for whom and in what contexts interventions work (C, context), the underlying processes that cause outcomes (M, mechanisms), and the nature and extent of the outcomes (O, outcomes). Realist theories are usually framed as CMO hypotheses. Our primary research question was: “In which situations, how, why, and for whom does SDM between patients and health care providers contribute to improved engagement in the Shared Decision-making process?” That is, what are the contexts and mechanisms that lead to better engagement in SDM? To address this question, we explored:

1. What mechanisms can facilitate or hinder engagement in the SDM process?
2. What contexts can affect the expression of the identified mechanisms?
3. In which contexts do varying mechanisms apply?

By identifying CMOs, we developed a program theory articulating the main mechanisms that result in successful, or unsuccessful, SDM during medical consultations. While previous papers have identified facilitators and barriers to SDM, this manuscript links contextual factors with mechanisms to form hypotheses (in the form of CMOs), depicting how individuals may successfully engage in SDM.

## Methods

In this section we provide a brief review of the methodology used in this realist synthesis. A complete description of the methodology can be found in a previous publication [47].

### Realist Philosophy and Methodology

We chose realist methodology to understand the complexities of shared decision-making, identifying how, in which situations, for whom and why SDM works or fails. Realist methodology is grounded in the philosophy that contends the identification of generative mechanisms within programs. Realist philosophy acknowledges that the world is “real”, but the perception of the world is constructed through social and cultural interpretation [48], meaning that different mechanisms (and therefore different CMO configurations) operate for different people in varying situations their interpretation of the situation. Methods developed to be consistent with its philosophical underpinnings means that realist methodology is specifically useful for examining complex interventions [12] and program nuances.

Developed by Ray Pawson, realist syntheses examine how and why an intervention is successful or fails [12, 48, 49]. This is done by identifying, in the existing literature, the mechanisms (forces or processes, commonly invisible, causing change), the contexts in which they do and do not operate, and the outcomes they generate [48–50]. Realists develop middle-range theories in the form of context-mechanism-outcome (CMO) configurations, forming testable hypotheses. Middle-range theories at are a level of abstraction that describe how a program, or particular aspects of it, is thought to work [51]: the theories are abstract enough to apply across contexts but specific enough to derive testable hypotheses from them. Hypotheses can also draw on substantive, or formal theories, and the evidence previously collected about them. Use of formal theories to support CMO configurations helps connect reasoning to the interactions between context and mechanism. Together, this forms a program theory that defines how outcomes occur. A program theory is therefore a collection of CMO configurations, supported by formal theories, depicting testable hypotheses.

To conduct our realist synthesis, we followed the realist synthesis process developed by Pawson [49], and visually depicted by Molnar, adding stakeholder involvement [47, 52] in accordance with the Realist and Meta-narrative Evidence Syntheses: Evolving Standards (RAMESES) realist training guidelines [50, 53, 54]. This included seven steps: (1) preliminary program theory development, (2) search strategy development, (3) selection and appraisal of literature in accordance with realist methodology (1, 2), (4) data extraction, (5) identification of relevant formal theories, (6) data analysis and synthesis, and (7) formation of a revised program theory with the input of stakeholders. We consulted with stakeholders as part of our seventh step to ensure the program theory reflected their experiences with the decision-making process. This process has been described in detail in our previously published methodology paper [47].

### Preliminary program theory development

Our team conducted a scoping review of literature [32], and drafted a *preliminary program theory* with the initial results (Additional file 1). This *preliminary program theory* began at the point of the patient accessing health care (or choosing to not access health care), and continues on to follow the patient through the SDM process and demonstrated primary and secondary outcomes of SDM implementation. This preliminary program theory is intended to act as an initial sketch of our understandings of SDM to form hypotheses for our formal search strategy. Following this process to explicitly state our rough understanding without a formal synthesis of SDM follows training methods outlined by RAMESES [50].

### Search strategy

**Fig. 1:**
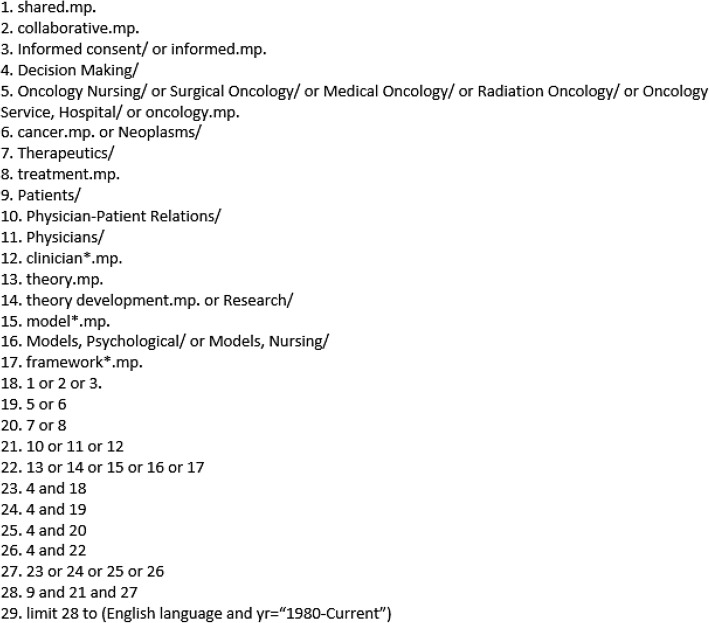
Purposive Search Strategy. This figure depicts the original purposive search strategy undertaken for this synthesis

We began with a purposive (targeting SDM specific literature) search of the SDM literature through PubMed and Google Scholar using the following keywords: “shared”, “collaborative”, “decision-making”, “informed”, “oncology”, “cancer”, “treatment”, “patient(s)”, “physician(s)”, “clinician(s)”, “theory”, “development”, “model(s)”, and “framework(s)” (Fig. 1). Oncology was utilized as an exemplar of SDM because it demonstrates a complex decision-making process. However, we did not exclude studies that fell outside of an oncological scope. Snowball sampling and an expanded scoping examination of a secondary search were performed through Medline (Additional file 2). The secondary Medline search reflected revisions from our primary strategy (for example the inclusion of health care providers beyond the physician), and reflects the iterative nature of realist research. Snowball sampling included searching highly cited sources, as well as documents from key researchers in the area (such as Francé Légaré and Dawn Stacey) [55]. Following RAMESES guidelines, we utilized grey literature within our search which was sought from health jurisdictions [53]. All literature searching was completed by three authors, TW, TC and VD.

Screening was completed by two team members using the following inclusion criteria: (1) an exchange between a patient and/or patient’s family and a health care provider, (2) a clinical situation where the patient is legally competent to make his/her own decision, (3) adult patients (18 years or older) making decisions about their own medical situation, (4) sources from 1980 to present, (5) English-language sources only (due to the language constraints of our team). 1980 was chosen as this is when SDM was first popularized in research. Empirical, theoretical, or grey literature were all included. Studies were excluded if their focus was on surrogate decision-making, when patients are unable to be involved (e.g., end-of-life care, pediatric decision-making, reduced competency, and dementia), or if they did not assess factors that attributed to SDM engagement of the health care provider and patient (such as papers that focused on clinical outcomes of SDM). Pawson’s criteria of relevance and rigor were also used to assess each source for applicability and methodological appropriateness, respectively [50]. Sources were excluded on account of rigour if they did not demonstrate validity and verifiability of findings (scientific articles only) or transparency [50, 56].

### Data extraction

Each source was read in full and explanatory accounts (EA) statements were extracted, in the form of ‘if-then’ statements (formulated as: if “x” occurs in “y” context, then “z” will be the result). An extraction template was used for this process which included: 1) bibliographic information; 2) notes relevant to the developing program theory; 3) country of study/document; 4) focus of document (patient and/or HCP); 5) empirical or theoretical (if relevant). Any middle-range theories that were used were noted by the authors within this template. EA statements were extracted from the results and discussion segments of papers, often connecting multiple findings within a single statement. A list all EA statements has been previously published within a thesis document [32].

### Stakeholder session: composition and analysis

Our stakeholder session was comprised of knowledge users who had experience with medical decision-making. Recruited through the local health region and provincial health ministry, this group was composed of two oncology patients, two nurse navigators, one oncology specialist, one family physician, and two policy makers. Patients were recruited through a provincial patient forum with a self-identification process. Stakeholders were identified as those who would be most impacted by the implementation of SDM, as indicated in literature. Two individuals from each stakeholder category were chosen to allow adequate representation. The semi-structured session lasted three hours and participants were asked their perspective on whether the program theory represented their experiences. The session guide has been previously published [32]. Data gathered from this session were used to refine the program theory (see stakeholder results section).

In-session notes and the session transcript (transcribed from audio-recordings) were analyzed using retroductive techniques, as per realist evaluative processes [49]. Retroductive analysis identifies demi-regularities and mechanisms that drive an outcome, while discounting mechanisms that do not appear to have causal powers in a specific context of interest [57]. Using NVivo 11, one researcher on our team (TW) identified demi-regularities within the transcript and coded whether stakeholders confirmed, suggested refinement, or refuted any of the program theory. This was done by comparing demi-regularities in stakeholder transcripts to contexts, mechanisms, and outcomes in the initial program theory. Once analysis was completed our team discussed and used the findings to refine the program theory. Further details on this process can be found in our previous publications on this project [32, 47].

### Expert consultation

**Fig. 2:**
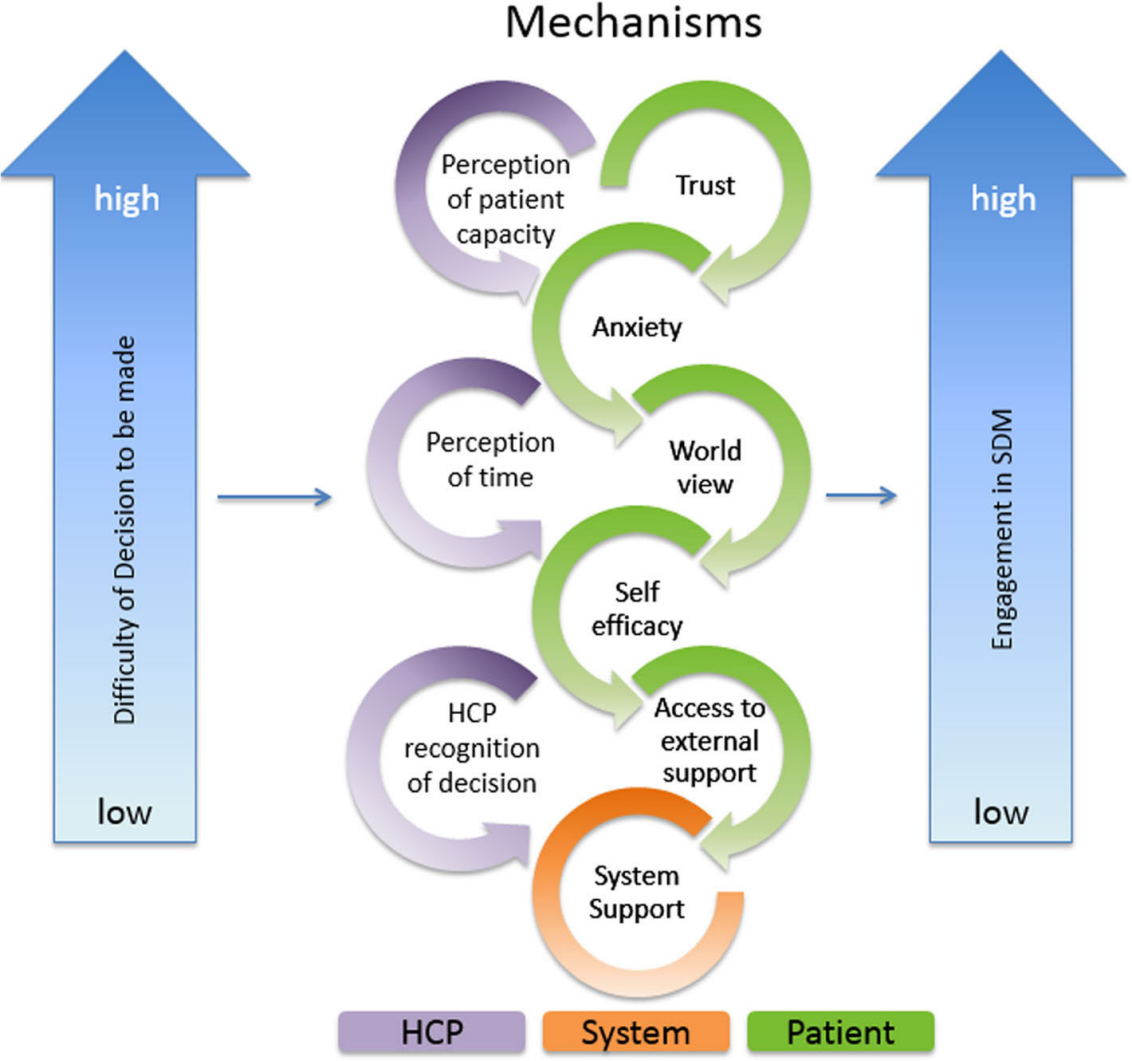
Initial Program Theory. This figure depicts our Initial Program Theory, which was shown to stakeholders

To ensure our program theory was in accordance with SDM principles, we presented our *initial program theory* (Fig. 2) to Dr. France Légaré, the Canadian Chair of Shared Decision-making and Knowledge Translation, a co-principal investigator of the IP-SDM model, and her team (ten internal and six members representing a hospital in Denmark implementing SDM). Experts were chosen based on their expertise in the area as well as the geographical accessibility to the authors. We implemented recommendations from this discussion to revise the final outcome from “mutually agreed upon decision” to “patient-centered and informed decision” to reflect current terminology in the field.

## Results

### Search results with selection and appraisal of documents

**Fig. 3:**
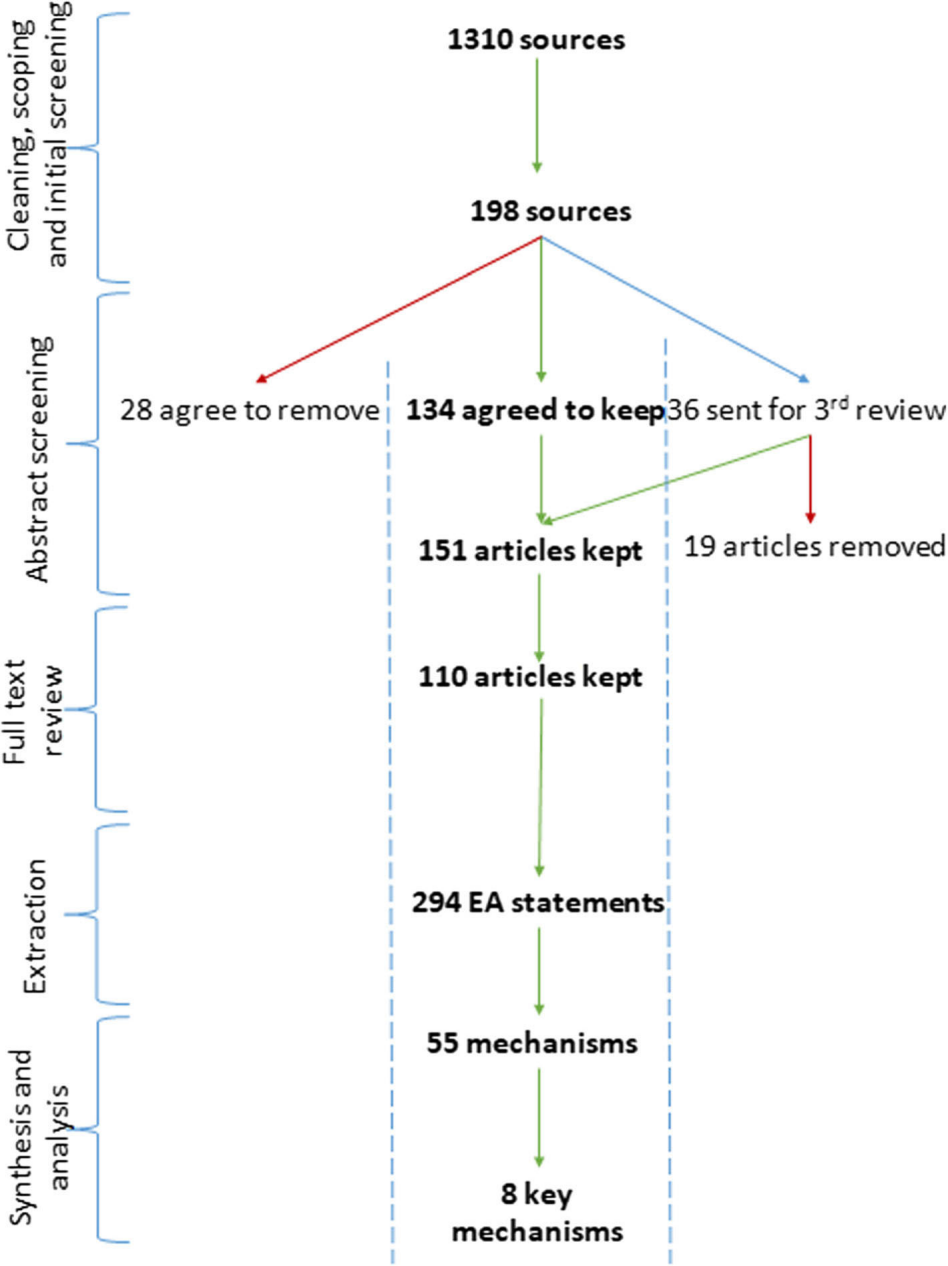
Screening and Synthesis process. This figure depicts the screening and synthesis process that authors undertook to achieve the final eight key mechanisms

Our searches resulted in 1310 references which were screened by title and abstract. From this, 198 documents underwent full-text review and 110 articles remained after final screening (Fig. 3). The articles were highly concentrated in North America and Europe, but represented the following countries: Australia (2), Belgium (1), Canada (28) Europe – General (1), France (1), Germany (3), Multi-country (6), Netherlands (1), Norway (1), Spain (2), Sweden (5), United Kingdom (15), United States (41), and Wales (6).

### Data extraction

Data extraction was conducted by TW and TC. A total of 294 EA statements were formed as a result of this process. We did not identify complete middle-range theories in the literature (i.e., included all elements to create a full CMO configuration). Typically, EA statements were composed of two-thirds of the CMO configuration (i.e., a context and mechanism, a context and outcome, or a mechanism and an outcome). An example of this process (using an excerpt from Maffei, 2012 [58]) can be found in Additional file 3.

### Analysis and synthesis

We created four categories to identify who the EA statements were specifically targeting: health care professionals (*n* = 180); patients (*n* = 374); both health care providers and patients (*n* = 81); and health care system factors (*n* = 10). Within these four categories, EA statements were placed into a thematic group. Researchers derived themes based on demi-regularities found within the extracted EA statements. This process of consolidation formed a total of 61 thematic groups under the four target categories. If an EA statement could fit into more than one thematic group (e.g. patient anxiety and patient trust), it was placed into both thematic groups. This caused an inflation in the total count of EAs within each category, compared to the total number of extracted EA statements.

Once all EA statements were consolidated our research team examined each thematic group to identify CMOs. A total of 55 mechanisms (Additional file 4) were identified specific to health care provider (33), patient (17), health care provider-patient interaction (3), and health care system (2). Members of our team then identified contexts and outcomes relevant to each mechanism to form CMO configurations, based on the initial extraction data.

We further analyzed the list of 55 mechanisms to isolate ones we considered ‘key mechanism sets’. Key mechanism sets are mechanisms that fall under a single label (for example, anxiety) but include mechanisms that can inhibit or facilitate SDM dependent on context. Based on our clinical (GG, LM), patient (LB) and research (literature (TC, TW)) expertise, the entire team identified nine mechanisms believed to hold the most causal power, or sets of mechanisms, which we hypothesize are critical for how the process of SDM works or fails. Table 1 names the *initial* nine key mechanisms that we identified at this stage. The identification of all mechanisms, formation of CMO configurations, and the identification of key mechanisms was done at an extensive multi-day team workshop. With the aggregated CMO configurations, we were able to develop and visually depict our program theory.

**Table 1:** Preliminary key mechanisms. This table outlines the nine key mechanisms that were originally identified by our team following data synthesis. These mechanisms were incorporated into the initial program theory and presented to stakeholders for confirmation, refinement, and refutation

Health care provider recognition of need for decision	
Health care provider and patient preference/willingness for engagement	
Health care provider perception of patient competency/capacity	
Health care provider perception of time available and required for SDM	
Patient anxiety	
Patient capacity to access external support and information	
Patient belief in their ability (self-efficacy) to participate in SDM	
Health Care System Support (including decision aids)	
Patient trust in individual health care provider as a person and as a professional	

### Revised program theory

A *focused IP-SDM mechanism map* (Additional file 5) which included only the key mechanism sets (*n* = 9) was then created. The *focused IP-SDM mechanism map* incorporated mechanisms that can hinder and/or facilitate engagement in SDM (that is, engagement in SDM was the outcome of interest). A pathway of SDM is visually depicted based upon the IP-SDM pathway [17]. IP-SDM was chosen as the base model as we believe it exemplifies many positive criteria of SDM, including the involvement of multiple parties. Mechanisms placed above the SDM pathway are those that may facilitate engagement, whereas those below may hinder engagement. However, our program theory acknowledges that mechanisms may work to either hinder or to facilitate depending on context. We determined how mechanisms facilitated or hindered SDM based on the formed CMO configurations from our analysis of synthesized CMO statements.

To better demonstrate the level of complexity within our program theory, we developed our *initial program theory* focusing on only the key mechanisms (Fig. 2). Based on our analysis of the literature, key mechanisms interact in a fashion that prohibited them from being untangled from one another. The *initial program theory* recognizes difficulty of decision as an important context that interacts and influences how various key mechanisms are triggered. Dependent on how an individual assesses the complexity of the diagnosis, mechanism sets will trigger in varying gradients. For example, if a patient received a diagnosis with complex implications (such as cancer), that context may trigger a higher level of anxiety. Similarly, remaining key mechanisms will be variably expressed; a complex decision is thought to influence the extent to which other key mechanisms are also triggered. Together, the combination of how the context and mechanisms manifest will determine the level of engagement the HCP and patient can achieve, impacting the decision that is made. Therefore, each of the key mechanism sets in the *initial program theory* have their own gradient, which may shift in expression in each consultation, producing different levels of engagement. Together the interaction within the key mechanisms (how mechanisms impact one another when expressed) and context results in a varying level of engagement in the SDM process from both the patient and HCPs. When engagement in SDM is of a high quality from both the patient and HCP, it then enables a patient-centered and informed decision.

### Formal theory of decision-making

Throughout the data extraction phase, TW and TC extracted any formal theories that were used within the SDM literature to underpin the decision-making process. A few theories were identified from our sources; however, only three (Theory of Planned Behaviour (ToPB), Feeling of Rightness (FOR), and Expected Utility Theory) were able to assist in the explanation of the developing middle-range theories. An extension of the Theory of Reasoned Action, the ToPB depicts the volition an individual has, or does not have, to control a decision [59], determined by one’s attitude, subjective norms, and perceived behavioural control. Upon completion of the revised program theory, a second theory - Feeling of Rightness (FOR) [60] - gained the attention of our research team. The Feeling of Rightness is defined as an individual having a strong intuition of being correct (e.g. suitability of a specific treatment option), causing the individual to no longer search for an answer or alternative reasoning [60, 61]. A third theory, Expected Utility Theory, was identified from health care decision-making literature [62, 63]. This formal theory indicates that individuals will try to make a decision based on what they assess will have the most favourable outcome in the future, given a set of actions, contexts and corresponding outcomes [64]. The Expected Utility Theory describes the impact uncertainty has on a decision [62] based on the utility of potential outcomes. Applying this to our PT, when the complexity of a disease increases, a patient is likely to experience higher uncertainty in what will provide them the best outcome.

### Stakeholder sessions

Stakeholders were shown our *initial program theory* (Fig. 2). Stakeholder perspectives were elicited on the following: 1) if the program theory matched their experience (or did not), 2) if the program theory matched their ideal decision process, 3) if the identified mechanisms were the most important, and 4) if program theory terminology resonated with the group.

Stakeholders were generally supportive of the *initial program theory*; however, some key points of refinement were suggested. One point was to add additional key contextual factors, to reflect the potential impact of pre-existing relationships between patients and health care providers. Relationship contexts could either facilitate or hinder the process of SDM. If a physician has a positive professional history with the patient (context), the physician may accurately assess the patient’s preferred level of engagement which would promote the process by increasing patient trust. However, if the HCP makes incorrect assumptions, or if a negative history exists, this may increase patient anxiety and decrease patient trust. The second key point was that the stakeholders believed that the key mechanisms could apply to both patients and health care providers, rather than being separated by role within the consultation (i.e., exclusively HCP or patient).

Stakeholders and researchers continued to explore the role of the key mechanisms, during which we decided to revisit the literature regarding health care system support to better understand how it acts as a mechanism. This iterative fashion of analysis is in-line with the nature of realist research, and the formation of program theories [50, 53]. At this point, it was determined that our results did not have enough evidence to validate system support as a mechanism. However, our analysis did indicate system support was a context underpinning the engagement process, therefore, we re-categorized system support to represent a context rather than a mechanism, resulting in eight key mechanisms or mechanism sets. While we are able to identify that aspects of system support (such as availability of decision aids) could directly impact the mechanism of perception of time from the HCP perspective, further research into the nuances of contexts is required. For example, the availability of decision aids provided systematically to HCP (context of system support) may create a perception of increased time requirements (mechanism) to guide the patient through the aid, potentially resulting in a negative impact on the HCP’s decision to use SDM. Thus it is important for further investigation into system support to determine contextual components that impact key mechanisms. We further verified the categorization of remaining contexts and mechanisms based on our data analysis at this time, which resulted in no further changes.

**Fig. 4:**
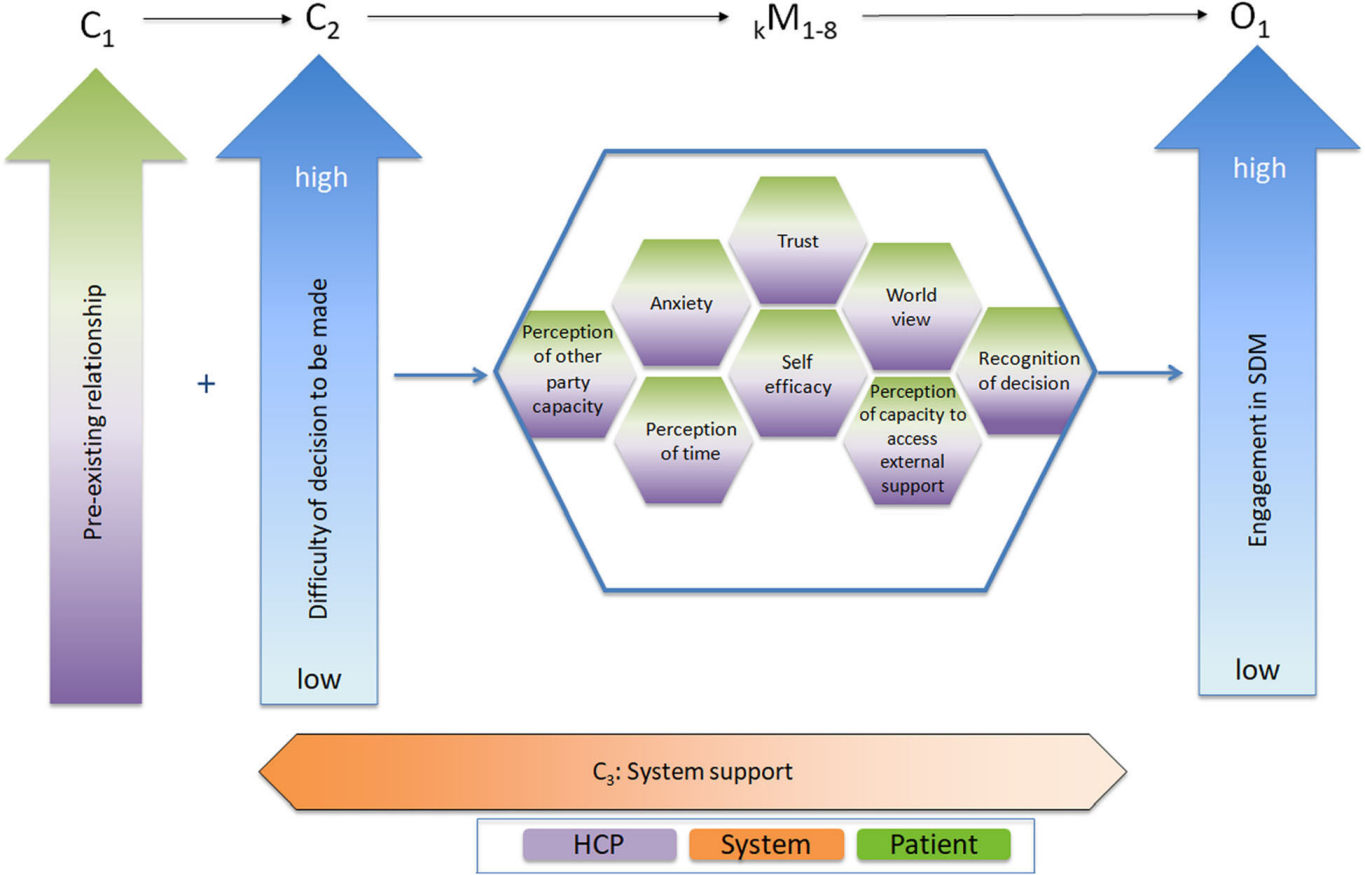
Revised Program Theory. This figure represents our Revised Program Theory, beginning with the nature of any pre-existing relationship and difficulty of decision to be made. These interacts with the key mechanisms, while the context of system support continues throughout process. Together, the contexts and mechanisms form the level of engagement within SDM

**Fig. 5:**
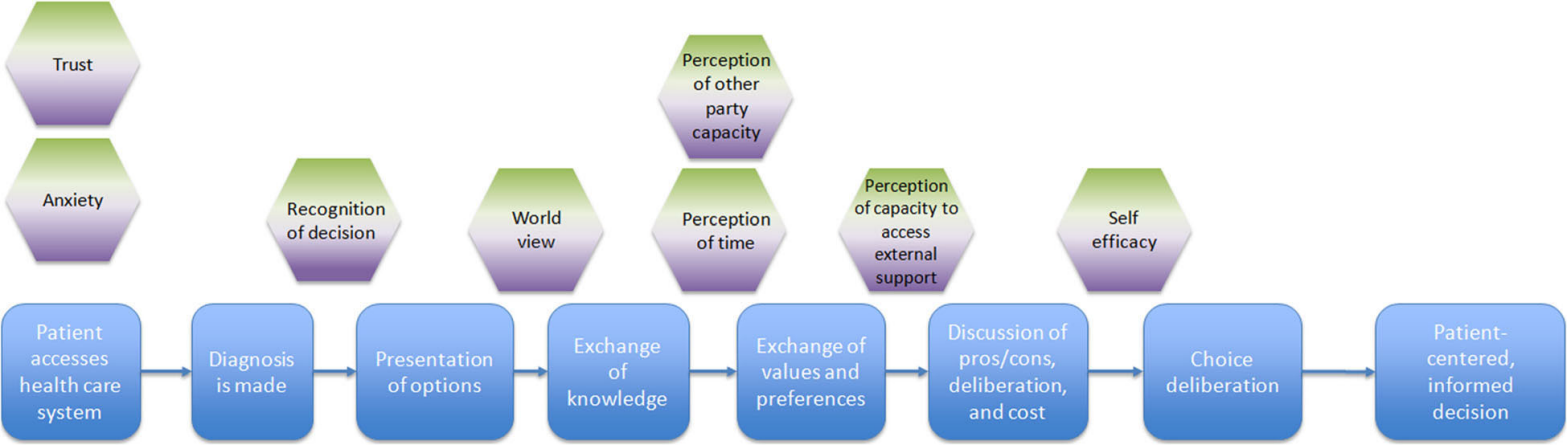
Revised Focused IP-SDM Mechanism Map. This figure overlays the IP-SDM steps (blue) with the identified key mechanisms of the process. Here, mechanisms are aligned with the area they are thought to first manifest in the process

Following analysis of the stakeholder session, our team made a final refinement to form our *revised program theory* (Fig. 4). The adaptation of the *initial program theory* included stakeholder perspectives: the inclusion of the additional context (pre-existing relationship between patient and HCP), and the adaptation of all mechanism sets to be relevant to the patient and the HCP. The white space within the honeycomb pattern represents the existence of other mechanisms, such as the remaining 45 mechanisms identified in our analysis that were not included as key mechanisms. These key mechanisms interact with other elements of the contexts, to determine the level of engagement in SDM during the consultation. Finally, we returned to our *focused IP-SDM mechanism map* and implemented the changes from the stakeholder session, creating the *revised focused IP-SDM mechanism map* (Fig. 5).

### Summary of revised program theory

Our *revised program theory* contains three contexts, eight key mechanism sets, and an outcome. As there are certainly additional contextual factors that impact specific mechanisms, it is important to note that further research into the contexts relevant to the identified mechanisms is required. The three contexts are pre-existing relationship, difficulty of decision and health system support. The key mechanism sets have been labeled as: perception of other party capacity, anxiety, perception of time, trust, self-efficacy, world view, perception of capacity to access external support, and recognition of decision. Each labeled set represents a concept that was identified in the literature that may positively or negatively impact the outcome of interest, but for which there presumably exists two or more mechanisms at play, depending on the concept and in which direction its impact is. However, the interactions in and between these mechanism sets are nuanced and literature gathered did not provide enough depth to fully elucidate all necessary details for full understanding. All key mechanism sets manifest in both the patient and the HCP. Finally, the outcome remains engagement in SDM. The visual representation of our *revised program theory* (Fig. 4) was formed based on the interplay between CMOs that we identified during the analysis. For example, the context of pre-existing relationship was placed at the beginning of the figure as our analysis indicates it may influence how a patient perceives his or her disease at time of initial diagnosis. Table 2 discusses the definitions of each segment of the program theory, sorted alphabetically. Table 3 further depicts CMO configurations for key mechanisms. These CMO configurations demonstrate testable hypotheses that one may use to predict SDM engagement. For example, if there is a high level of difficulty in the decision (context) and the patient has high anxiety (mechanism), then there will be low patient engagement in SDM (outcome). Therefore, it is possible to predict the outcome of low engagement based on disease severity and patient anxiety.

**Table 2:** Definition of concepts in the Revised Program Theory

	Factor	Definition
Key Mechanisms	Anxiety	The level of worry or nervousness felt before or during the consultation. This can be specifically related to the decision process/diagnosis, or other outside influences.
Key Mechanisms	Perception of capacity to access external support	The perception of the individual^a^ in relation to their ability to obtain support outside of the consultation. This can include, but is not limited to: support groups, family and friends, colleagues, internet resources, and manuscripts.
Key Mechanisms	Perception of other party capacity	The perception held by one individual regarding the other’s ability to successfully meet the expectations of their role within the consultation. For example, the perception the patient has regarding the HCPs knowledge and experience for their disease.
Key Mechanisms	Perception of time	The perception of how long it takes to implement SDM, and the amount of time available for the consultation. HCPs may perceive inadequate time allotted to implement SDM. This can include potential time pressures on the patient. This also incorporates the perception of time available to make a decision (e.g. perceived urgency of treatment).
Key Mechanisms	Self-efficacy	The individual’s belief he/she is able to participate within the SDM process. For example, whether the HCP believes he/she can successfully exchange their knowledge and expertise to the patient, and whether the patient believes they can adhere to potential treatment options. This may also be influenced by whether a healthcare system has provided appropriate supports for patients and HCPs to successfully implement SDM.
Key Mechanisms	Trust	The level of trust and confidence that the individual feels for the other person. For patients, this also includes the trust for the HCP as a professional. For HCPs, it may include the trust for patient to adhere to the treatment or be forth-coming.
Key Mechanisms	World view	The set of beliefs, customs, values, morals and/or understandings that the individual holds about the medical process that may align with, or clash against, biomedical definitions of health care. This may incorporate aspects such as religion and culture.
Key Mechanisms	Recognition of decision	Whether the HCP or patient consciously acknowledges that a decision-choice exists.
Context	Difficulty of decision to be made	The perception the individual holds on the how complex the decision needing to be made is. This can be significantly affected by values and preferences, as well as experience.
Key Mechanisms	Pre-existing relationship	The existence, duration, and quality, of a professional relationship between the patient and the HCP(s) prior to the consultation. This may also include assumptions that may be made based on the nature of the pre-existing relationship.
Context	System support	The presence of policy, training, financial, decision tools, and managerial support for the implementation and use of SDM within consultation. This can extend to the extended time allotment for consultation and providing decision tools, among other supports.
Outcome	Engagement in SDM	The degree to which the individuals, together and individually, are able to cohesively engage within the SDM process given the interaction of key mechanisms.

This table presents the contexts, mechanisms, and outcomes that are incorporated into the revised program theory, and definitions of each concept. These definitions represent what was found in our synthesis; future research may highlight the need for modification ^a^individual is operationally defined as either the health care professional and/or the patient

**Table 3:** Descriptions of key mechanisms in the Revised Program Theory

Mechanism Category	Detailed CMOs
Anxiety	Facilitation of SDM: 1. If a patient faces a moderately difficult decision regarding their treatment, then they may experience a moderate increase in their anxiety fostering a drive to engage within SDM.Moderate difficulty of decision (C) + Moderate anxiety (M) ➔ Patient engages in SDM (O) 2. If a HCP has received system support to gain skills in SDM, then they may have reduced anxiety about using it within their consultation, increasing their engagement in SDMSystem support for SDM (C) + Reduced anxiety (M) ➔ HCP engagement in SDM (O) Hindering of SDM: 1. If a physician perceives high patient anxiety, then they may unilaterally decide it is inappropriate to engage in SDMPatient displaying high anxiety characteristics (C) + HCP perception of patient anxiety (M) ➔Low engagement in SDM by the HCP (O) 2. If a patient has a difficult decision regarding their treatment, then they may experience a debilitating increase in anxiety resulting in low patient engagement in SDMHigh difficulty of decision (C) + High patient anxiety (M) ➔ Low patient engagement in SDM (O)
Perception of capacity to access external support	Facilitation of SDM: 1. If a HCP perceives that the system offers supports to aid in the decisional process, then they are more like to engage in SDM.Perception of System support (C) + Perception of capacity to access external support (M) ➔ high engagement in SDM (O) 2. If a patient believes that they have supports beyond the HCP, then they are likely to experience reduced anxiety and increased self-efficacy, resulting in high SDM engagementPerception of capacity to access external support (C) + Reduced anxiety (M) + Increased self-efficacy (M) ➔ High SDM engagement (O) Hindering of SDM: 1. If the HCP is dealing with a complex diagnosis and does not perceive that they are able to access external support such as journal articles, then they are likely to experience low self-efficacy in SDM and have reduced SDM engagement Complex diagnosis (C) + Perception of capacity to access external support (M) + Low self-efficacy ➔ Low SDM engagement (O)
Perception of other party’s capacity	Facilitation of SDM: 1. If HCPs have received the appropriate training through their system, then they are able to adjust their SDM approach based on their perception of patient capacity, increasing HCP engagement and improving the patient’s ability to engage in SDM. System support (C) + Accurate perception of patient capacity (M) ➔ High patient and HCP engagement in SDM (O) Hindering of SDM: 1. If a patient is displaying high levels of anxiety, then the HCP may perceive that they do not have the capacity to participate in decision-making, resulting in a low HCP engagement in SDMHCP perception of patient anxiety (C) + HCP perception of patient capacity (M) ➔ Low HCP engagement in HCP (O)
Perception of time	Facilitation of SDM: 1. If the HCP perceives they have system support to give patients as much time as they require for decision-making, then the HCP and patient will have a higher level of engagement in SDMSystem support (C) + Perception of time (M) ➔ High engagement for HCP and patient in SDM process (O) Hindering of SDM: 1. If a system is set for a fee-for-service schedule – which does not incorporate consultation time appropriately into the schedule – and the HCP perceives that SDM increases appointment times, the HCP may elect to reduce their time spent with the patient, negatively impacting the HCPs level of engagement. Negative system support for SDM (C) + Perception of inadequate time to conduct SDM (M) ➔ Low HCP engagement in SDM (O) 2. If a HCP perceives that a decision must be made immediately, they may not engage the patient as they do not perceive the time to incorporate their opinions. As an example, if an individual comes in with a life-threatening emergency, the HCP is more likely to act without patient consultation.High complexity of diagnosis (C) + Perception of limited time to make a decision (M) ➔ Low engagement of SDM by the HCP, limiting patient engagement (O) 3. If a HCP believes that they do not have the flexibility within their schedule (e.g., case load, system support to appropriately consult, etc.), they may elect to not involve, or inadequately involve, the patient in the decision process. Low system support (C) + Perception of inadequate time available (M) ➔ Low SDM engagement (O)
Self-efficacy	Facilitation of SDM: 1. When the patient is able to express their preferences and values through the implementation of SDM, then they experience higher confidence in their ability to participate in SDM, resulting in higher levels of SDM engagement. System support for SDM use (C) + Increased patient self-efficacy (M) ➔ High engagement in SDM (O) Hindering of SDM: 1. If an individual (HCP or patient) does not believe they are capable of participating in SDM, then they will avoid attempting engagement.Unidentified context + Low self-efficacy (M) ➔ Low engagement in SDM (O)
Trust	Facilitation of SDM: 1. If a patient trusts the HCP (or a HCP trusts the patient), then they will engage in SDM. Pre-existing relationship (C) + trust (M) ➔ high engagement in SDM (O) 2. If the HCP perceives that the patient trusts them, then the HCP will engage in SDM. Unidentified context + perceived trust (M) ➔ high engagement in SDM (O) Hindering of SDM: 1. If a patient does not trust the HCP (or a HCP does not trusts the patient), then they will not engage in SDM. Pre-existing relationship (C) + Lack of trust (M) ➔ Low engagement in SDM (O)
World view	Facilitation of SDM: 1. If a HCP is willing to incorporate the patient’s world view of the biomedical model into the treatment options, then the patient more likely to engage in SDM. For example, patients may not wish to explore certain treatment options (such as blood transfusions) based on their world view. *HCP acceptance of world view*^*a*^ (C) + World view (M) ➔ High SDM engagement (O) Hindering of SDM: 1. If a HCP is not willing to incorporate the patient’s world view of the biomedical model into the treatment options, then the patient unlikely to engage in SDM.*HCP un-accepting of world view*^*a*^ (C) + World view (M) ➔ Low SDM engagement (O)
Recognition of decision	Facilitation of SDM: 1. If a diagnosis is complex and requires a lot of information exchange, then HCPs are more likely to recognize that the patient must be involved in the decision and SDM engagement increases.Complex diagnosis (C) + Recognition of decision (M) ➔ SDM engagement (O) Hindering of SDM: 1. If a HCP recognizes that a decision is required to be made, then SDM engagement will occur.Unidentified context (C) + Recognition of decision requirement (M) ➔ SDM engagement (O)

This table presents the CMOs for each mechanism set identified within the program theory. ^a^italics represent hypothesized contexts

## Discussion

As far as the authors are aware, this research is the first realist synthesis of SDM literature. This synthesis creates a program theory which identifies the mechanisms that facilitate, or hinder, SDM implementation. This paper has outlined eight key mechanism sets for SDM engagement; anxiety, trust, world view, perception of time, self-efficacy to engage in SDM, perception of capacity to access to external support, recognition of decision, and the perception of capacity of the other party. These key mechanism sets interact with one another and with varying contexts to help explain when SDM may work (or not work), for whom, in what circumstances, and why. We overlapped these key mechanisms with the IP-SDM model to understand when these factors may become important during the decision-making process.

Our *revised program theory* offers a novel understanding of how SDM works, for whom, in which circumstances, and why or why not. Specifically, it demonstrates that SDM works in a complex manner, and for any individual patient and HCP there may be an array of interconnected mechanisms at play. Further, these mechanisms can change in their expression continually depending on contexts. We have built upon previous research by linking facilitators and barriers to contextual factors which alter how mechanisms are expressed. While we aimed to form a program theory that clearly uncovered how SDM works, our analysis uncovered mechanisms that trigger variably based on the simultaneous expression of partnering mechanisms. This has resulted in the formation of testable hypotheses to investigate in future works, and has initiated the first step towards understanding the nuance within SDM.

Our findings align with previous studies of SDM facilitators and barriers. Through a systematic review Joseph-Williams and colleagues (2014) identified eight factors that patients reported could potentially inhibit their involvement in SDM, giving specific attention to the power imbalance between health care professionals and patients [65]. The authors conclude that this may translate into patient anxiety, and the power imbalance likely changes depending on the nature of the pre-existing relationship. This is congruent with our findings that anxiety and pre-existing relationships are key mechanisms in the SDM process. Further, this is likely reflected in whether the physician consciously recognizes the need for a decision, and for the patient to be involved in this decision. Gravel and colleagues (2006) also examined factors that promote and hinder SDM from a clinical perspective in their systematic review [66]. They identified that physician self-efficacy and perception of time to implement SDM were inhibitors to the process. They also listed physician world view as a motivator, in terms of HCP beliefs that SDM would improve patient outcomes. While these articles both identified barriers and facilitators, neither examined the entirety of the process (i.e., patient, health care provider, and system factors), and did not indicate how these barriers and facilitators interacted within the SDM process. Without developing theory based on how both barriers and facilitators impact the process of SDM it is difficult to predict when such factors play a role, in what situations, and how.

### Practical implications

Our results have several potential practical implications. Health care professionals can use this program theory to identify key areas to focus on to provide patient-centered care. Through this, HCPs may be able to identify patients that require increased support based on those who display concerning levels of factors identified in our analysis, such as high anxiety. We anticipate that the program theory will help increase clarity and understanding within the complexity of patient care. Similarly, policy makers may be able to identify where system changes, such as increased consultation times or additional training and education, may be necessary.

Our *revised program theory* may be used convey the complexity of SDM to current and future HCPs. Guided by this theory, HCPs could better understand how to approach consultations with an array of patients, and gain increased capacities to successfully, and appropriately, attend to the key mechanisms. This program theory makes SDM more explicit by depicting how pre-identified barriers and facilitators influence the SDM process. It will encourage HCPs to provide education to patients in specific areas, and indirectly increase the quality of patient care.

### Formal theories

We identified three formal theories that together describe how the program theory can be used to determine engagement in the SDM process: the Theory of Planned Behaviour (TOPB), Feeling of Rightness (FOR), and Expected Utility Theory. While none of these theories individually explains how engagement in SDM occurs through our revised program theory, the three formal theories support segments of the program theory – such as specific mechanisms, contexts, or predicting the overall engagement outcome. A description of how these formal theories link to the program theory, and a description of their impact, can be found in Table 4. In brief: the TOPB predicts an individual’s behavioural intention through attitude toward behaviour, subjective norms, and perceived behavioral control [59]. TOPB supports seven of the key mechanisms, as well as overall engagement in SDM, by explaining how individuals form intent for an action. This is made through an individual’s assessment of a situation and the likely outcomes of producing a certain behaviour. We have applied the FOR to five of the key mechanisms to assist in explaining how they interact with the SDM process. FOR underpins these mechanisms in one of two ways: a strong feeling of rightness or a weak one. When a strong response to a stimuli or event is made an immediate heuristic response occurs, while a weak response will cause an individual to reformulate their position, which includes cognitive capacity to do so [61]. That is, an individual is likely to follow their intuition when they have a strong sense that it is correct, while a weak intuitive sense will cause the individual to think through their decision more completely. Finally, Expected Utility Theory underpins the context/intermediate mechanism of difficulty of decision through its theoretical description of how individuals react to unknown probabilities [62]. This is achieved by linking the amount of complexity in the decision to the ability for an individual to determine the utility, or positive future impacts, based on the potential options. In situations where complexity is increased, this theory would indicate that the uncertainty would increase in a positively correlated fashion. However, while we have identified these as formal theories underpinning our program theory, future research is required to investigate these hypotheses.

**Table 4:** Substantive Theories Underpinning the Revised Program Theory. This table presents the substantive theories that are incorporated into the *revised program theory*, and believed to underpin the SDM process

Formal theory	Area of program theory which the theory underpins	Impact of theoretical underpinning on SDM
Theory of Planned Behaviour (TOPB)	Anxiety, Trust, World view, Self-efficacy, Perception of capacity to access external support, Pre-existing relationship, Recognition of Decision, Engagement in SDM	The TOPB combines one’s attitude toward behaviour, subjective norms of the individual, and the individual’s perceived behaviour control to form the individual’s intention to conduct a certain behaviour. In SDM, someone can enter a consultation process with a predetermined idea of how they foresee the process going, and it can bias the success of the engagement process. For example, one may have norms engrained in their world view that create the behavioural intent to disengage from Western medicine, thus blocking the engagement process.
Feeling of Rightness (FOR)	Trust, World view, Self-efficacy, Perception of other party capacity, Pre-existing relationship	Patients and health care providers will make an initial assessment based on their previous knowledge and similar experiences from which they will conclude a feeling of rightness based on the fluency of recall, familiarity, and metacognitive beliefs. This will cause an individual to either accept their initial judgement or re-evaluate.
Expected Utility Theory	Difficulty of decision	If outcome probabilities of a given treatment are known, then individuals will have an easier time engaging with the decision-making process than if the impact is uncertain.

### Limitations

Our research may have been impacted by publication bias (research supportive of SDM is more likely to have been published than that refuting it). To mitigate this, we examined grey literature in medical decision-making. Due to the amount of available literature, and the resources of our team, our search was focused on the medical decision-making rather than decision-making literature more broadly. Future research could be undertaken to broaden the scope. In addition, the time taken from initial searching in 2015 to the refinement of our program theory following stakeholder analysis in mid-2017 may have allowed for newer information to be released. To alleviate the potential impact, we continually investigated new literature, through Ovid literature updates on our secondary search.

Realist methodology acknowledges that there will always be other interpretations of data, and final knowledge is never achievable [48]. We are not attempting to capture all nuances and complexity of SDM, as it is not feasible, but rather we focus on key mechanisms of the process. We have strived to extract mechanisms to build the most explanatory program theory from the literature by using multiple sources of information: literature, clinical expertise, and stakeholder consultation. Through the use of these multiple sources, we were able to limit interpretation bias on the part of the researchers.

A related, but important limitation to our work is that we identified three contexts that impact the manifestation of all mechanisms, rather than identifying mechanism specific contexts. As this project focused heavily on identifying the key mechanisms, future research is needed to explore what other contexts play a role in this process. This work is required to form a more complete understanding of how the mechanisms operate for different people and in different situations. However, we believe that this synthesis has undertaken the first step in identifying and understanding contexts and mechanisms that impact SDM engagement.

### Future research

Our program theory is novel in revealing the complexities of SDM. We acknowledge this as the first step to a series of applications of our findings. We encourage future research to test the program theory towards determine its applicability within different contexts. This process will confirm, refine, and/or refute the program theory in whichever context it may be applied. Specific examination in additional contexts (e.g. cultures, public or private care, etc.) may uncover additional key mechanisms. The program theory can be adapted to specific populations for a broader understanding of how engagement within SDM can occur.

Our team has begun exploring decision-making in a few areas. One area is with the Saskatchewan Indigenous population, and how culture and beliefs influence their decision-making process, and the resulting implications for the program theory. Using interviews with Indigenous patients living with cancer, and a focused realist synthesis on Indigenous world view and trust, we are refining the program theory to reflect the decision-making experience of the Indigenous populations. We have also conducted focused testing of the program theory in the context of prostate cancer patients. We anticipate testing the program theory in different areas to confirm, refine, and/or refute these findings, creating a more fine-tuned program theory for specific populations.

## Conclusion

Using a realist synthesis, and following RAMESES guidelines, we have conducted a realist synthesis of SDM. To our knowledge, this is the first examination into the mechanisms of SDM and how they inhibit and facilitate SDM implementation through patient and health care provider engagement. We have produced a program theory depicting key mechanism sets that can inform health care professionals to tailor their consultation process to each individual patient, and can provide a tool for policy makers to identify necessary system level changes. These findings allow us to better understand “In which situations, how, why, and for whom SDM between patients and health care providers contributes to improved decision-making”.

## Supplementary information

**Additional file 1.** Preliminary Program Theory. This depicts the preliminary program theory that was developed by the authors following an initial scope of the SDM literature, based on the understanding of how SDM worked and the outcomes of implementation.

**Additional file 2.** Refined Medline Search Strategy. Refined Medline Search Strategy Secondary search strategy, conducted October 16th, 2015.

**Additional file 3.** Example of Data Extraction, Synthesis, and Analysis process. An example of how article texts were extracted into EA then synthesized into thematic grouping and eventually analyzed to CMO configurations.

**Additional file 4.** All Identified Mechanisms. All identified mechanisms extracted from EA statements.

**Additional file 5.** Focused IP-SDM Mechanism Map. A mechanism map connecting key mechanisms set IP-SDM.

## Data Availability

The datasets used during the current study are available from the corresponding author on reasonable request.

## Abbreviations

C: Context
CMO: Context mechanism outcome
EA: Explanatory account
FOR: Feeling of Rightness
HCP: Health care provider
IP-SDM: Interprofessional-Shared Decision-Making
M: Mechanism
O: Outcome
RAMESES: Realist and Meta-narrative Evidence Syntheses: Evolving Standards
SDM: Shared Decision-making
ToPB: Theory of Planned Behaviour
