# Supplementary figures and images for: Development of a program theory for shared decision-making: a realist synthesis
Source: BMC Health Serv Res. 2020 Jan 23;20:59. doi: 10.1186/s12913-019-4649-1 (PMC6979294; doi:10.1186/s12913-019-4649-1)

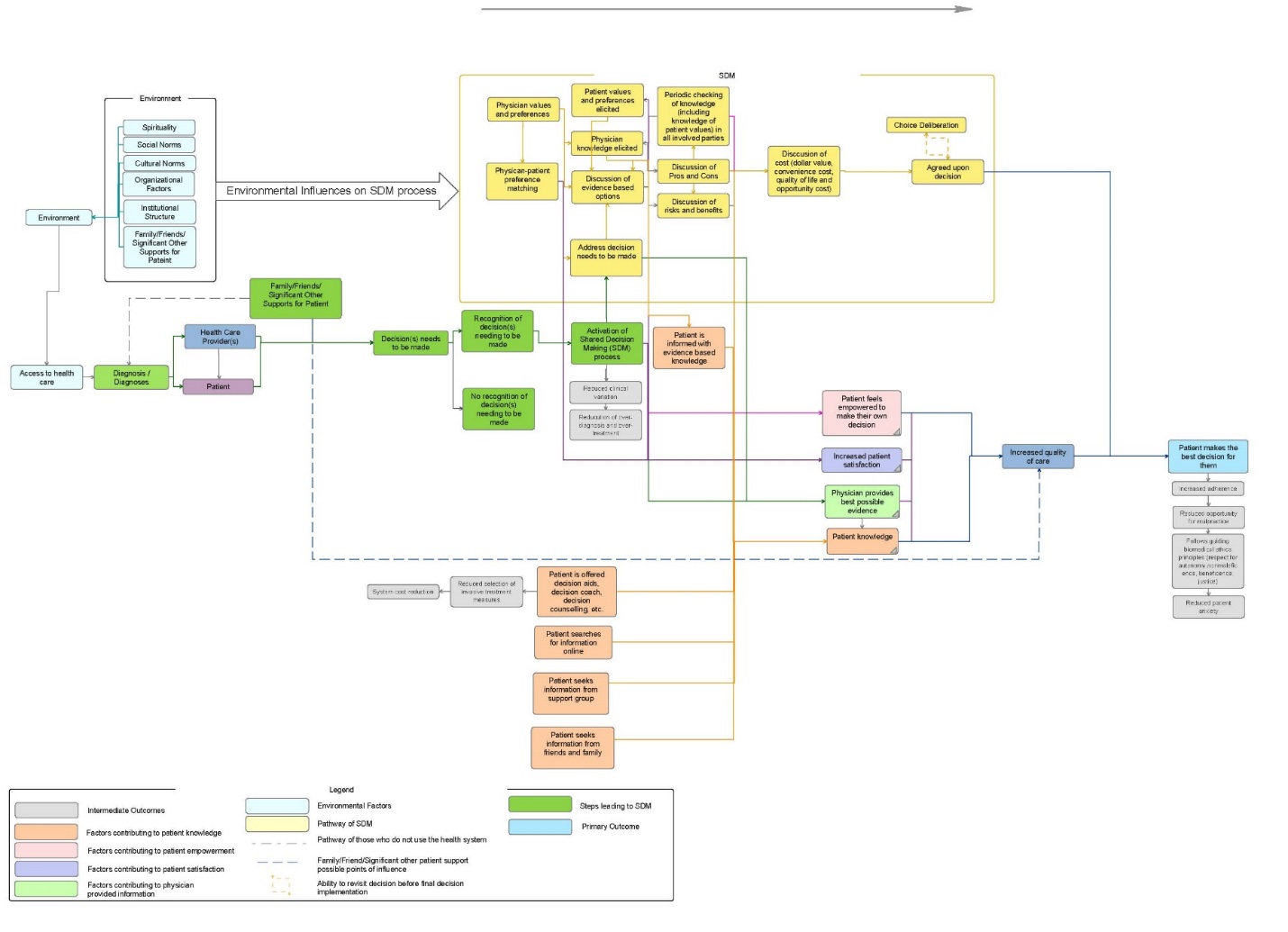

Supplement: Supplementary file 1 — Additional file 1. Preliminary Program Theory. This depicts the preliminary program theory that was developed by the authors following an initial scope of the SDM literature, based on the understanding of how SDM worked and the outcomes of implementation. [file 12913_2019_4649_MOESM1_ESM.docx]

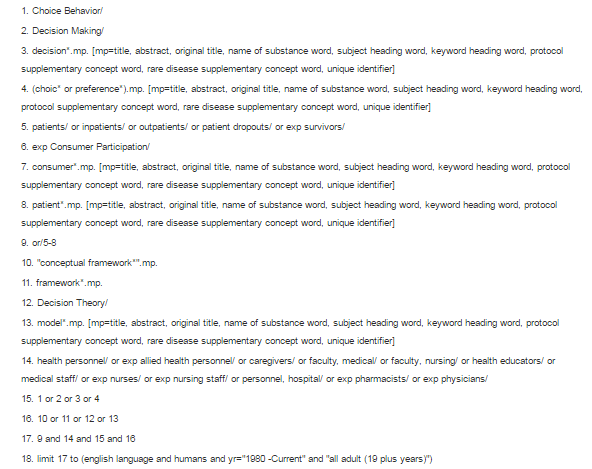

Supplement: Supplementary file 2 — Additional file 2. Refined Medline Search Strategy. Refined Medline Search Strategy Secondary search strategy, conducted October 16th, 2015. [file 12913_2019_4649_MOESM2_ESM.docx]

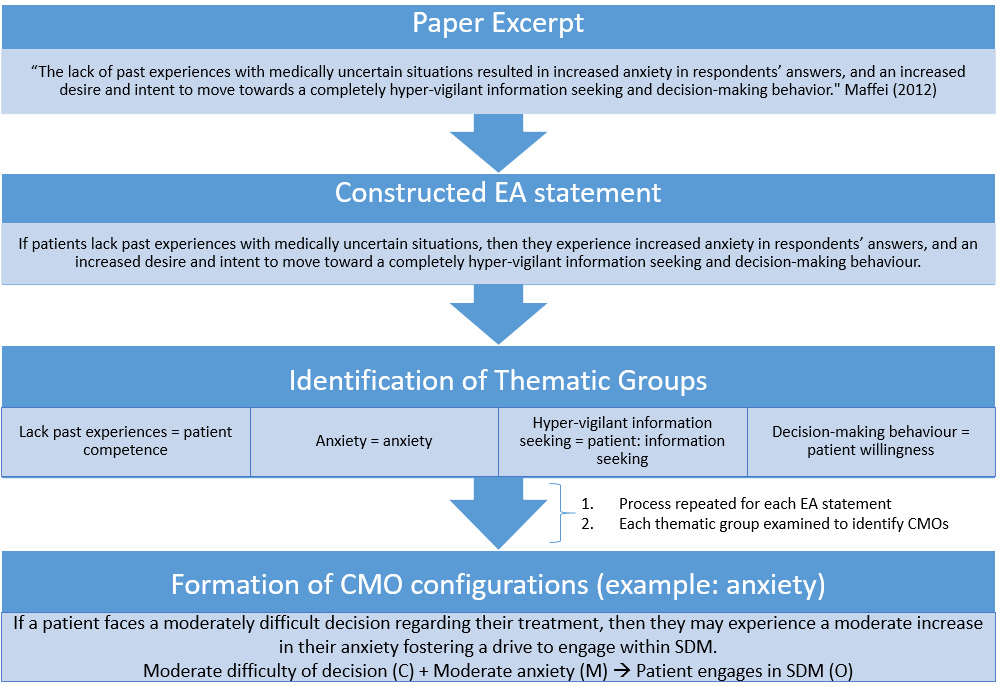

Supplement: Supplementary file 3 — Additional file 3. Example of Data Extraction, Synthesis, and Analysis process. An example of how article texts were extracted into EA then synthesized into thematic grouping and eventually analyzed to CMO configurations. [file 12913_2019_4649_MOESM3_ESM.docx]

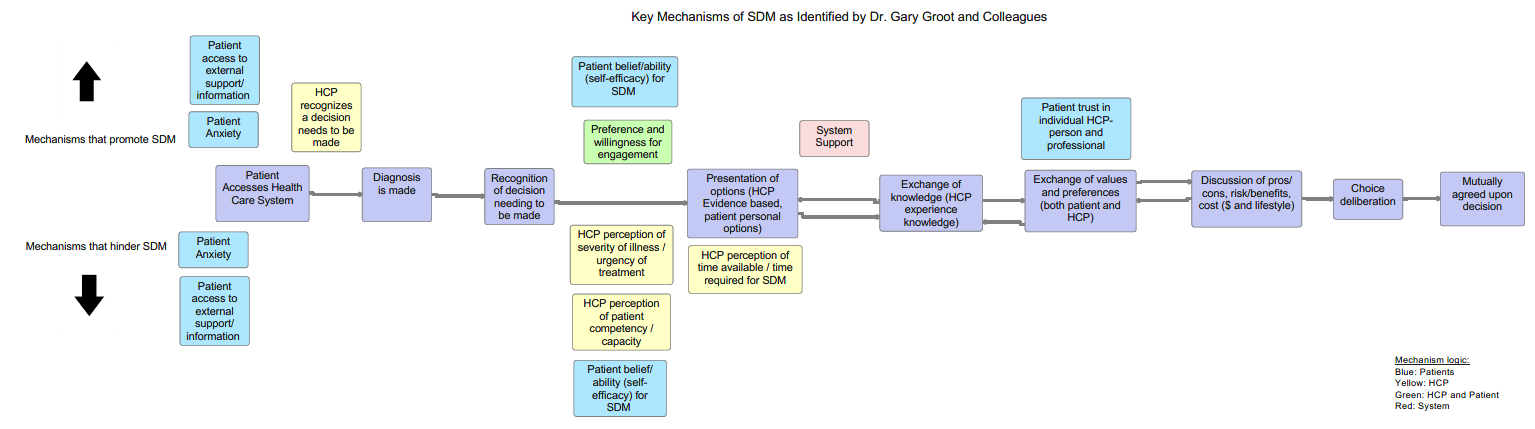

Supplement: Supplementary file 5 — Additional file 5. Focused IP-SDM Mechanism Map. A mechanism map connecting key mechanisms set IP-SDM. [file 12913_2019_4649_MOESM5_ESM.docx]
