# Supplementary material for: Development of a program theory for shared decision-making: a realist synthesis
Source: BMC Health Serv Res. 2020 Jan 23;20:59. doi: 10.1186/s12913-019-4649-1 (PMC6979294; doi:10.1186/s12913-019-4649-1)
Supplement: Supplementary file 4 — Additional file 4. All Identified Mechanisms. All identified mechanisms extracted from EA statements. [file 12913_2019_4649_MOESM4_ESM.docx]

| **Health Care Provider Specific Mechanisms** |
| --- |
| HCP perception of patient competency/capacity |
| Physician perception of severity of illness/urgency of treatment |
| Physicians perception of patient as too ill to make good decisions |
| Physician perception of older pts feelings of respect for profession |
| Physician belief that patient is anxious |
| Physician perception of patient trust |
| Physician perception of patient preference for involvement/engagement |
| Physician perception of patient support (external) |
| HCP perception of treatment options |
| HCP perceive treatment impacts pts lifestyle/self-image |
| Physician perception of likelihood of compliance + treatment |
| Physician perception of quantity and quality of information available |
| Physician perception of workload |
| Physician perception of time available <-> time required for SDM |
| Physician perception of level of effort involved in training programs |
| HCP comfort with implementing SDM |
| Physician concern about reimbursement |
| Physician support of patient involvement |
| Physician belief that patient autonomy should be respected but not enforced |
| Physician considers FIFE - feelings, ideas, function, expectations of PATIENT |
| Physician perception in the effectiveness of SDM |
| HCP has perception of value of patient centered care |
| HCP perceives patient as equal |
| HCP considers patient values/preferences |
| HCP recognize a decision needs to be made |
| HCP perceive they don't know how to determine SDM |
| Physician emphasis of importance of information exchange |
| HCP preference for engagement |
| Physician perception of appropriateness of SDM for situation |
| Physician competence <-> ability to “hear” patient preference |
| Physician dissonance: prior beliefs interfere with ability to accept and understand info |
| Fear of prosecution / loss of credentials |
| Decreased HCP anxiety |

| **Patient Specific Mechanisms** |
| --- |
| Patient perception of lack of power/ not being taken seriously |
| Patient sense of being respected |
| Patient trust in individual HCP-person + professional |
| Patient perception of physician disclosure |
| Patient fear of backlash / upset physician |
| Patient perception of own knowledge |
| Alignment between eventual decision and patient values/aspirations |
| Patient efficacy for SDM |
| Patient capacity to understand and process information (diagnosis, options, etc.) |
| Patient dissonance: prior beliefs interfere with ability to accept and understand info (Cultural beliefs / world view) |
| patient anxiety |
| Fit between patient desire for participation <-> experience in “this shared decision” experience |
| Patient preference for engagement |
| Patient desire for information |
| Patient desire to please – good patient – compliance |
| Patient expectations of physician |
| Increased treatment efficiency |

| **Health Care Provider – Patient Interaction Specific Mechanisms** |
| --- |
| Physician – patient communication – physician ability and willingness to bring the patient capacity gap |
| HCP and PT enhanced awareness and education of SDM |
| Perception of partnership (authentic and constructive) |

| **System Specific Mechanisms** |
| --- |
| Influence of training environment on HCP |
| System support |
